# *In vivo* measurement of human brain material properties under quasi-static loading

**DOI:** 10.1098/rsif.2022.0557

**Published:** 2022-12-14

**Authors:** Nicholas J. Bennion, Stefano Zappalá, Matthew Potts, Max Woolley, David Marshall, Sam L. Evans

**Affiliations:** ^1^ School of Engineering, Cardiff University, Cardiff CF10 3AT, UK; ^2^ School of Computer Science and Informatics, Cardiff University, Cardiff CF24 3AA, UK; ^3^ Cardiff University Brain Research Imaging Centre (CUBRIC), School of Psychology, Cardiff University, Cardiff CF24 4HQ, UK; ^4^ Functional Neurosurgery Research Group, School of Clinical Sciences, University of Bristol, Bristol, UK; ^5^ Renishaw Neuro Solutions Ltd, Wotton Road, Wotton-under-Edge GL12 8SP, UK

**Keywords:** brain, material properties, *in vivo*, stereotactic neurosurgery, finite-element analysis

## Abstract

Computational modelling of the brain requires accurate representation of the tissues concerned. Mechanical testing has numerous challenges, in particular for low strain rates, like neurosurgery, where redistribution of fluid is biomechanically important. A finite-element (FE) model was generated in FEBio, incorporating a spring element/fluid–structure interaction representation of the pia–arachnoid complex (PAC). The model was loaded to represent gravity in prone and supine positions. Material parameter identification and sensitivity analysis were performed using statistical software, comparing the FE results to human *in vivo* measurements. Results for the brain Ogden parameters *µ*, *α* and *k* yielded values of 670 Pa, −19 and 148 kPa, supporting values reported in the literature. Values of the order of 1.2 MPa and 7.7 kPa were obtained for stiffness of the pia mater and out-of-plane tensile stiffness of the PAC, respectively. Positional brain shift was found to be non-rigid and largely driven by redistribution of fluid within the tissue. To the best of our knowledge, this is the first study using *in vivo* human data and gravitational loading in order to estimate the material properties of intracranial tissues. This model could now be applied to reduce the impact of positional brain shift in stereotactic neurosurgery.

## 1. Introduction

Finite-element (FE)-based computational models of the human brain are an increasingly common research tool, with applications ranging from head impact to neurosurgery. Studies considering head impacts are generally concerned with traumatic brain injury (TBI), where a better understanding of the underlying mechanisms is essential for the development of prevention measures [1]. Within neurosurgery, efforts are primarily focused on tumour resection, where loss of cerebrospinal fluid (CSF) and tissue resection are responsible for much of the deformation [2]. Movement and deformation of the intact brain, known as brain shift, is clinically significant in stereotactic neurosurgical procedures such as deep brain stimulation where electrode placement accuracy correlates with patient outcomes [3]. Procedures such as convection enhanced delivery require submillimetre precision in placing catheters in the brain [4], navigating on pre-operative magnetic resonance imaging (MRI) and computed tomography (CT) images alone. Several studies have shown that re-orientation of the head leads to clinically significant displacements of the deep brain [5–8]. The aim of this study was to understand better the mechanics of this process through computational modelling, and hence to improve the outcomes of stereotactic procedures through pre-operative prediction of brain shift. Identifying the material properties of the brain through non-invasive *in vivo* measurements may also be valuable for many other applications.

For the brain, significant strain-rate dependency of the material properties exists. At high strain rates, there is little time for fluid flow and the material response is nearly incompressible [9]. As a result, very nearly incompressible or volume preserving viscoelastic material formulations for the brain are often used [1,10,11]. Low-strain-rate applications such as neurosurgery may employ poroelastic constitutive models if the time-dependent stress state is desired. These typically employ consolidation theory [12], analogizing brain tissue as a fluid-soaked sponge, where loading redistributes fluid through the solid matrix over time [13], manifesting as local compressibility of the otherwise incompressible tissue. This approach adds complexity to the mathematical representation and requires identification of additional material parameters [14]. When strains are small, equilibrium can be reached within minutes [8], meaning the time-dependent state may be unimportant for quasi-static loading. In such cases, use of an elastic, compressible material to represent the drained state after fluid redistribution may be an appropriate simplification.

Mechanical testing of human brain has numerous challenges. Regional heterogeneity means that samples are generally limited in size [15]; the extremely low stiffness of brain leads to collapse under its own weight, making preparation of the sample to a precise, small geometry even more challenging. The stiffness of the tissue changes once removed from the body, accentuated by changes in temperature [16], or the use of preserving agents such as formaldehyde. Multiple tests on the same sample have shown a significant pre-conditioning effect, which recovers after soaking the sample in fluid [15]. This shows that the role of interstitial fluid redistribution is significant. However, subsequent calculations of the material properties often consider the tissue to be incompressible, not accounting for fluid movement during the test [15,17].

The apparent solution is to test the tissue *in vivo*, although there are clear ethical issues with performing invasive mechanical testing on live humans. Approaches such as shear wave elastography using ultrasound or magnetic resonance elastography are growing in popularity; however, these only derive the shear modulus at very low strains. Here, we intend to counteract the challenges of traditional measurement techniques by loading the brain through gravity alone, preserving the natural state of the tissue, and hence to identify the mechanical properties of the tissue, using our previously published MRI displacement data [8].

The most common approach to identifying material parameters is the ‘inverse FE’ or ‘FE model updating’ method where a model of the experiment is constructed and repeatedly solved in order to optimize the material parameters to minimize the difference between the model and the experiment. This approach requires repeated serial solution of the full nonlinear model, often thousands of times, and so it can be very slow. It also gives no indication of the uniqueness or uncertainty of the result or what range of other parameter values might be compatible with the experiment. Non-updating approaches include the virtual fields method, where full-field experimental measurements can be used to derive constitutive model parameters through the equivalency of internal and external virtual work [18]. An alternative approach is to run models for a range of parameter values (which can be done in parallel) and use statistical techniques to construct an emulator that predicts the model output. The emulator can then be used to evaluate a large number of parameter sets rapidly and hence to determine the range of potential values and the uncertainty in the identification process. Due to the size and complexity of the model, we used the latter approach, employing Gaussian process regression to eliminate the need for prohibitive numbers of computations.

## 2. Methods

### 2.1. Human study

To derive a high-resolution displacement field across the entire brain, a human MRI study was carried out in conjunction with Cardiff University Brain Imaging Research Centre (CUBRIC). Healthy subjects (*n* = 11, 7 male, 4 female) with a mean age of 25 years (range 22–30) were imaged. Full details of the study and data processing can be found in Zappalá *et al.* [8]. In brief, one prone image was taken after 20 min of pre-conditioning face down to ensure the brain had completely settled. Subjects were subsequently inverted to the standard supine position and scanned again. Structural T1-weighted MPRAGE sequences [19] were used to acquire images for all subjects, using Siemens 7T MAGNETOM (Siemens Healthcare, Erlangen, Germany) and Siemens 3T PRISMA scanners for subjects 1–8 and 9–11, respectively. To determine displacement across the cerebrum, the prone and supine images were first aligned using affine registration of the skull alone. Deformable registration was then performed from the prone to supine images, generating a vector displacement field over the entire volume in individual subject space. Finally, all subject displacement fields were normalized to the MNI 152 standard space [20].

### 2.2. Finite-element model design

**Figure 1.**
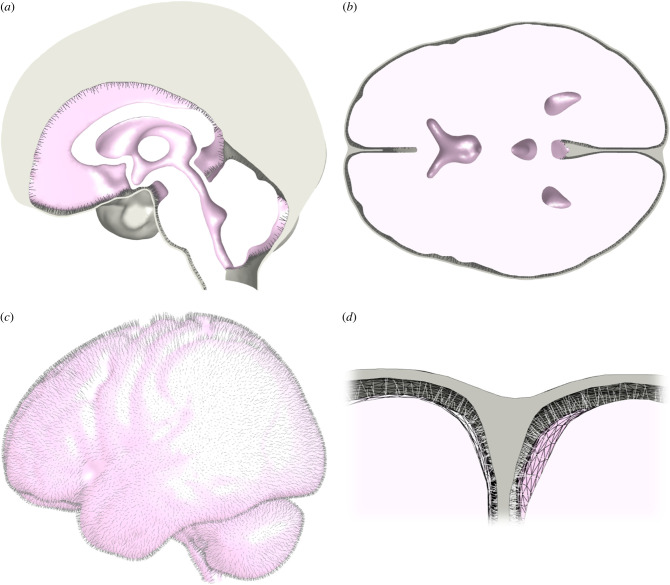
Final model geometry, depicting: a midline sagittal section (*a*), a transverse section at the approximate level of the anterior and posterior commissures (*b*), an exterior lateral view with the combined dural septa removed (*c*) and a sectioned view of the arachnoid trabeculae of the pia–arachnoid complex (spring elements) spanning the subarachnoid space (*d*). The CSF fills all internal voids within the model, but has been removed for clarity.

In order to recreate the desired anatomy while mitigating the impact of subject specific anatomical (as opposed to material) variations, the MNI 152 standard space [20] was also used as a base geometry for the FE model. Image segmentation was performed using Simpleware ScanIP (Synopsys, Mountain View, USA) and manually optimized to remove poor quality elements. As it is not possible to image the brain in a completely unloaded state, the subarachnoid space was segmented to have a minimum thickness of approximately 2 mm throughout. This was considered to approximate a ‘neutral’ position. The final segmented geometry consisted of three primary volumes: the brain, combined CSF filled space (figure 1*a*,*b*) and the combined dural septa (figure 1*a*,*b*,*d*). Volume meshing was performed within ScanIP using Tet4 elements. The final model contained 432 059 elements for the brain, 367 752 elements for the CSF and 376 309 for the dural septa.

FEBio [21] was chosen for FE analysis. Once the volume mesh was imported, the model file was modified using MATLAB (The MathsWorks Inc., Natrick, MA, USA) to generate the pia–arachnoid complex (PAC). The pia mater (figure 1*c*) was generated using two-dimensional shell elements sharing nodes with the surface of the brain, with a thickness of 15 µm [22]. Discrete spring elements were generated spanning the exterior surface nodes of the pia mater to interior surface nodes of the dura to replicate the arachnoid trabeculae (figure 1*c*,*d*), while preserving the subarachnoid space to be defined as a continuous fluid layer with the ventricles. The arachnoid mater was assumed to be adhered to the dura mater and therefore omitted.

### 2.3. Material representations

Variations of the Ogden constitutive model [23] are generally accepted to capture the response of brain tissue under multiple loading regimes [15,17,24]. A single term strain-energy density function *ψ* is commonly accepted [15] to represent the brain:

2.1 $$\psi =\frac{2\mu }{\alpha }({\bar{\lambda }}_{1}^{\alpha }+{\bar{\lambda }}_{2}^{\alpha }+{\bar{\lambda }}_{3}^{\alpha }-3)+U(J),$$

where ${\bar{\lambda }}_{1,}\;{\bar{\lambda }}_{2,}\;{\bar{\lambda }}_{3}$ are the deviatoric principal stretches. The distortional component contains parameters *μ* and *α* which represent the classical shear modulus [15] and strain stiffening response, respectively. The volumetric energy component is defined in equation (2.2):

2.2 $$U(J)=\frac{1}{2}k{\left(\ln J\right)}^{2},$$

where the volume ratio, *J*, is the determinant of the deformation gradient tensor **F**. In this case, *k* represents the apparent bulk modulus of the brain.

While the pia mater exhibits nonlinear behaviour [25,26], it was expected that strains would be small, and a neo-Hookean representation sufficient. The strain-energy density function of the neo-Hookean model in FEBio takes the form of equation (2.3):

2.3 $$\psi =\frac{\mu }{2}(I_{1}-3)-\mu \ln J+\frac{\lambda }{2}{\left(\ln J\right)}^{2},$$

where *μ* once again represents the shear modulus and *λ* the first Lamé parameter. *I*_1_ is the trace of the right Cauchy–Green tensor, **C**, where $C=F^{T}F$. Under small strains and rotations these reduce to classical linear theory based on Young's modulus, *E*, and Poisson's ratio, *ν*, as described by equations (2.4) and (2.5):

2.4 $$\mu =\frac{E}{2(1-\nu )}$$

and

2.5 $$\lambda =\frac{\nu E}{\left(1+\nu )(1-2\nu \right)}.$$

As the dura mater is orders of magnitude stiffer than the surrounding structures [27], it was considered a rigid body fixed to the interior surface of the skull. The arachnoid trabeculae were represented by spring elements, where the reaction force, *F*, of each spring was defined as shown in equation (2.6), where *a* is the spring constant and *x* is the scalar extension of the spring. The force is generated only when the spring is under tension, acting along the line of the spring upon the associated pair of surface nodes.

2.6 $$F=\{\begin{array}{c} 0,\;x\leq 0\\ -ax,\;x>0. \end{array}$$

The CSF was modelled as a Newtonian fluid [28], with fluid–structure interactions defined on the surfaces of the brain and combined dural septa. The viscous shear stress, ***τ***, is defined by equation (2.7) in terms of the shear viscosity, *μ* (not to be confused with the shear stress used previously), bulk viscosity, *κ*, and rate of deformation tensor, **D**. The fluid Cauchy stress, $\sigma^{f}$, is given by equation (2.8), where **I** is the identity tensor and *p* is the fluid pressure.

2.7 $$\tau =\left(\kappa -\frac{2}{3}\mu \right)(\mathrm{tr}\;D)I+2\mu D$$

and

2.8 $$\sigma^{f}=-pI+\tau .$$

Fluids are represented as a mixture of a fluid and a solid with negligible (but non-zero) stiffness. The solid phase defines the FE mesh and its stiffness helps to regularize the mesh as its boundaries deform. The fluid then flows through this mesh and the resulting pressures and shear stresses are coupled to the adjoining solids via a shared, continuous interface. A more complete explanation of the governing equations is available in the FEBio User's Manual [29].

One of the main objects of this study was to identify the material parameters. This was undertaken through a parametric analysis, where the bulk modulus, shear modulus and stiffening coefficient of the brain material, Young's modulus of the pia mater and spring constant of the arachnoid trabeculae (PAC structure) were varied. Initial parameter ranges (Table 1) were derived from preliminary testing and the literature. The values included are limited to those present in fully converged models. Where only a high value is given, this parameter was kept constant and not considered as part of the parametric analysis.

**Table 1.** Material parameter value ranges used for the parametric analysis.

region	material model	parameter	low value	high value	units	source
brain	Ogden (uncoupled)	density	—	1040	kg m^−3^	[30]
		bulk modulus	14	1000	kPa	[12]
		shear modulus	0.5	1500	Pa	[15,17,24]
		exp. coef.	− 30	− 1		[15,17,24]
CSF	Newtonian fluid	density	—	1007	kg m^−3^	[31]
		bulk modulus	—	2.2	GPa	[32]
		shear viscosity	—	0.001^a^	mPa s	[33]
		bulk viscosity	—	2	mPa s	[33]
pia mater	neo-Hookean	density	—	—		
		Young's modulus	1	15 000 000	Pa	[25,26,34]
		Poisson's ratio	—	0.45		
arachnoid trabeculae	linear spring	spring constant	0.01	25	N m^−1^	preliminary testing
dura mater	rigid body					

^a^The shear viscosity for the CSF was set at an arbitrarily low value as the dynamic response was not desired.

### 2.4. Boundary conditions

Positive and negative body loads along the anterior–posterior direction of 9.81 m s^−2^ were applied to two separate models for each parameter combination, in order to replicate prone and supine orientations. The dura mater was fixed in all dimensions. The inferior surface within the foramen magnum (dura, CSF, spinal cord) was fixed in the inferior–superior axis only.

### 2.5. Computation and data processing

Due to the complexity of the model, computing every combination of parameters using traditional methods was not possible. Latin hypercube sampling [35] was used to generate 120 parameter sets, of which 105 successfully converged. The prone and supine models for each combination of parameters were computed with FEBio, using the Advanced Research Computing at Cardiff University (ARCCA) Hawk supercomputer. This system contains a Linux cluster of 7000 cores of Intel Skylake Gold 6148 processors (2.4 GHz/4.8 GB per core/20 cores per processor). Nodal displacements were exported as a text file; all subsequent data processing and analysis were performed using MATLAB.

The voxel displacement field of the human data was interpolated to the FE node space for each subject using trilinear interpolation. Data from the cerebellum were excluded due to intensity drop-off in the images, leading to highly variable displacement fields. Nodal displacements were averaged for each cerebral element (*n* = 361 031), yielding orthogonal element displacement components (*u_i_*, *v_i_*, *w_i_*). Each elemental displacement was then weighted by the corresponding relative element volume (*W_i_*, where *W_i_* = elemental volume/total volume) to remove nodal density effects. The ‘baseline displacement’ (*D_b_*), equivalent to mean displacement magnitude over the brain volume, was calculated according to equation (2.9) for each subject individually and the average of all subject displacement fields:

2.9 $$D_{b}=\overset{n}{\underset{i=1}{\sum }}\sqrt{{\left(W_{i}u_{i_{\mathrm{sub}}}\right)}^{2}+{\left(W_{i}v_{i_{\mathrm{sub}}}\right)}^{2}+{\left(W_{i}w_{i_{\mathrm{sub}}}\right)}^{2}}.$$

Equation (2.10) was used to calculate a scalar residual error value (*E_r_*) for each combination of computational model and subject data:

2.10 $$E_{r}=\overset{n}{\underset{i=1}{\sum }}\sqrt{{\left(W_{i}(u_{i_{\mathrm{sub}}}-u_{i_{FE}})\right)}^{2}+{\left(W_{i}(v_{i_{\mathrm{sub}}}-v_{i_{FE}})\right)}^{2}+{\left(W_{i}(w_{i_{\mathrm{sub}}}-w_{i_{FE}})\right)}^{2}}.\;$$

This residual error describes the level of displacement in the subject data unaccounted for by the computational model. The residual error was divided by the baseline displacement to give the ‘global error ratio’. This ratio captures ‘goodness of fit’ across the entire brain equally, therefore, representing the complex displacement pattern as a scalar which can be used for parametric analysis. The objective of the parametric analysis was to minimize this value.

A GPR model was used to estimate the residual error of untested parameter combinations. For initial parameter identification, the MATLAB ‘fitrgp’ function was used. The available 10 kernel functions were tested by training the model and subsequently predicting the residual error from the same parameter sets. The best performing function was ‘ArdSqauredExponential’ with a Pearson correlation coefficient of 0.999 between the input and predicted results. For use, the model was trained with the residual error of each of the 105 parameter sets and the average subject displacement. Residual error estimates were initially generated for 24 300 000 new parameter sets, representing every combination of parameters at 30 intervals between the minimum and maximum values given in table 1.

## 3. Results

**Figure 2.**
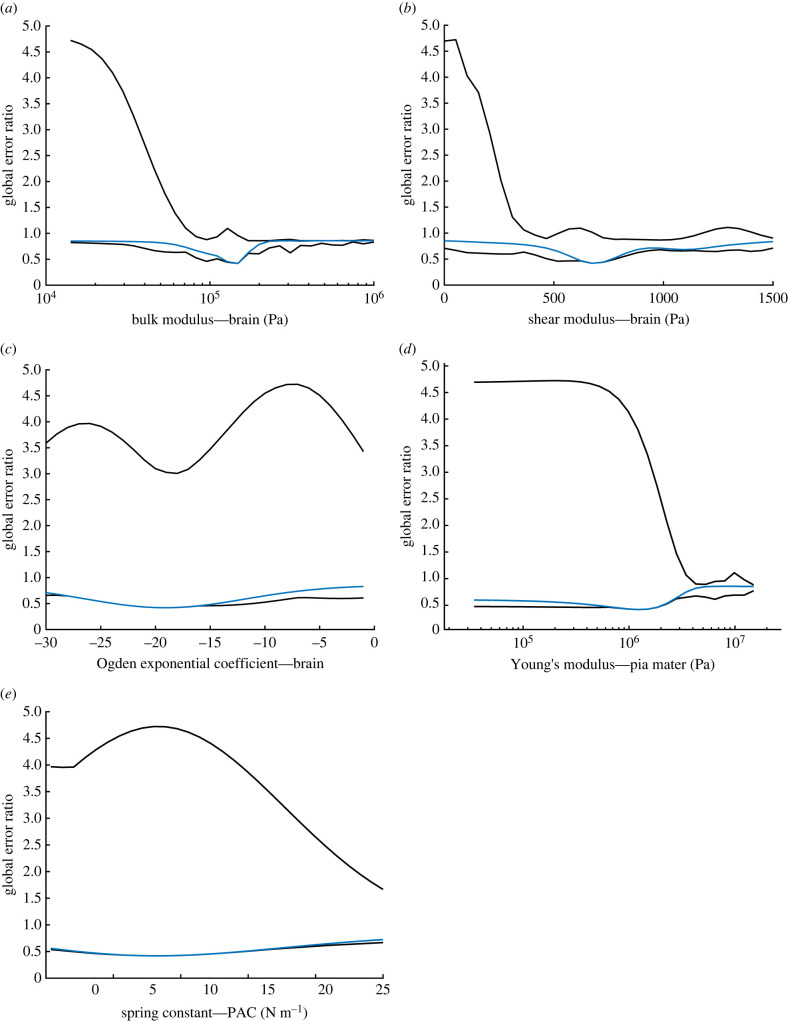
Plots depicting the minimum and maximum global error ratio change for the brain bulk modulus (*a*), shear modulus (*b*) and exponential coefficient (*c*), pia mater Young’s modulus (*d*) and PAC fibre stiffness (*e*) when varied across the ranges shown in table 2 (black). Overlaid is the variation in global error ratio (blue) based on variation of the individual parameter shown alone, with all others set to the optimum values. The minimum in each case represents the same set of parameters, representing the value at which the lowest error between subject data and computational model output was achieved.

The average of the 11 subject displacement fields was analysed first. For a detailed description of the displacement pattern within the human data, we direct the reader to our previous publication [8]. In brief, displacement was greatest in the deep brain, with peak magnitudes of the order of 1 mm, reducing to surface displacements typically less than 0.5 mm. Outputs from 24 300 000 combinations of input parameters were used to generate the high and low global error ratio estimates shown in figure 2 (black). These lines represent the best and worst combinations at each increment of the parameter shown.

The minimum of each plot shown in figure 2 represents the output from the ‘best’ combination of the five parameters tested. With the average displacement fields, these values were 148 kPa, 670 Pa, −19, 1210 kPa and 8.0 N m^−1^ for the brain bulk modulus (*k*), shear modulus (*μ*) and stiffening coefficient (*α*), pia mater Young's modulus (*E*) and PAC fibre stiffness (*a*), respectively. The baseline error of the averaged displacement field was 0.46 mm with a predicted residual error of 0.19. Figure 2 also demonstrates that some parameter sets led to levels of displacement greater than that in the subject data, with global error ratios up to approximately 4.5. The black curves represent the maximum and minimum possible levels of error predicted using any combination of the other parameters. Broadly, when the black curves have little separation, the other parameters are less influential and the ‘goodness of fit’ is largely determined by the parameter depicted. These curves demonstrate the potential impact of choosing arbitrary values for the other parameters. An indication of output sensitivity for each parameter is given in the overlaid plot showing output variation when all other parameters are set to the optimized values (blue). In these, a higher rate of change of error around the minimum broadly indicates greater sensitivity to that parameter. However, this must be interpreted carefully as the total range varies. To investigate this further, sensitivity analysis of the residual error was performed over the initial parameter ranges using free-to-use Gaussian Emulation Machine for Sensitivity Analysis (GEM-SA) software (available at www.tonyohagan.co.uk/academic/GEM). While implementing the same underlying principles, GEM-SA incorporates sensitivity analysis [36], more clearly demonstrating the influence of each input parameter on the output.

The total output variance was found to be 0.16 mm, of which 47.1% was accounted for by parameter change. Table 2 gives the percentage of the predicted output variance identified for each parameter alone (main effect) and the joint influence of every pair of parameters. The total effect combines these, to describe the influence of that parameter including higher order interactions. The bulk modulus and shear moduli of the brain were most significant, with total effects of 82% and 81%, respectively. The exponential coefficient of the brain, pia mater stiffness and PAC stiffness all had a lower impact on variance when considering the total effect alone, but only the pia mater remained unimportant when joint effects were considered.

**Table 2.** Summary of sensitivity analysis results, assessing the influence of each material parameter on the output residual error, considering both singular and joint effects for each.

	main effect						joint effect								
	*k*	*μ*	*α*	*E*	*a*	*k*.*μ*	*k*.*α*	*k*.*E*	*k*.*a*	*μ*.*α*	*μ*.*E*	*μ*.*a*	*α*.*E*	*α*.*a*	*E*.*a*
variance (%)	5.2	4.4	1.5	1.0	1.5	19.8	4.2	0.0	2.5	4.2	0.0	2.3	0.0	0.5	0.0
total effect (%)	82.0	81.0	51.2	1.0	37.4										

**Figure 3.**
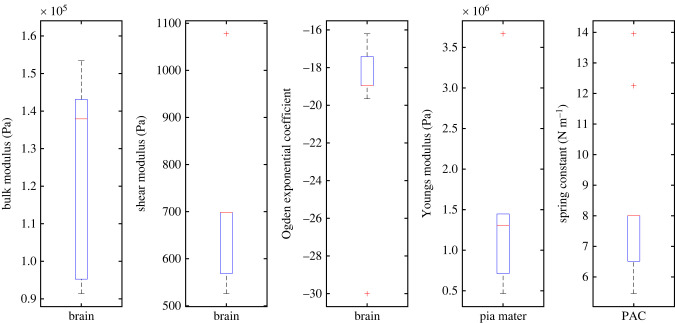
Boxplot showing the optimum values obtained for each of the 11 subject datasets for each tested parameter.

The parameter identification process was repeated with each of the subject datasets individually, with the tested ranges reduced in order to improve output resolution. Boxplots showing the derived optimum parameters from each of the 11 subjects can be found in figure 3. A full table of the individual subject results can be found in table 3 of appendix A.

Given that the spring distribution of the PAC was driven by the mesh density of the exterior surface of the brain, a conversion must be carried out to yield a comparable Young's modulus (*E*_PAC_). The conversion used is given in equation (3.1):

3.1 $$\;E_{\mathrm{PAC}}=\frac{n_{s}ax}{A_{t}},$$

where *n*_s_ is the total spring elements within the model, *a* is the spring stiffness, *x* is the mean initial spring length and *A_t_* is the surface area of the brain. At a spring stiffness of 8 N m^−1^, this equates to an approximate out-of-plane Young's modulus of 7.7 kPa for the PAC.

**Figure 4.**
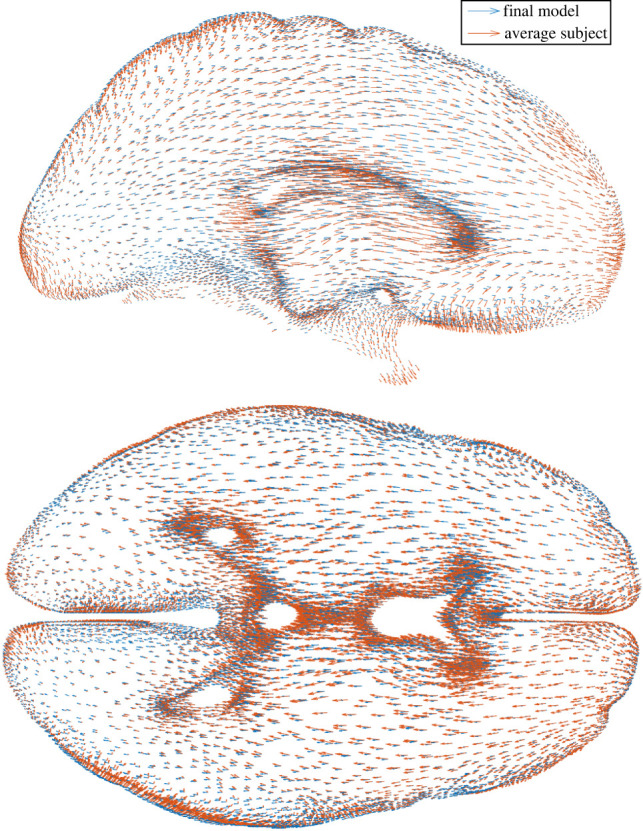
Displacement fields of the final model output (blue) and average subject dataset (orange) with prone-to-supine repositioning, demonstrated within 10 mm sagittal (top) and axial (bottom) sections.

The FE model was re-run with the optimized parameters in order to validate the predicted optimum parameter set. The results showed a residual error of 0.29 mm, slightly greater than predicted. Peak displacements in the FE model were around 0.5 mm, located within deep structures. Figure 4 shows a comparison of the FE and subject displacement fields. Within the subject data, boundary displacements appear to have a greater likelihood of being erroneous in direction and magnitude.

With the optimized material properties, it was possible to examine the biomechanics of prone to supine repositioning more closely. Deviatoric stress within the brain peaked at around 10 Pa, while volumetric stress peaked at around 1 kPa, varying linearly across the brain according to orientation. The resulting volume change across the brain was less than 1% with the optimized model; however, volume change in models with a high bulk modulus was negligible. Spring force was found to peak of the order of 0.2 mN in the posterior for prone positioning and anterior for supine, with the opposite side having little to no contribution. Stresses in the pia mater displayed the same trend, with peak stresses of the order of 1 kPa.

## 4. Discussion

### 4.1. Computational model

Developing the computational model was a laborious but essential step towards the subsequent analysis. Past modelling efforts have mainly focused on TBI [10,37–40] and large deformation neurosurgery [41,42]. Studies have considered stereotactic neurosurgery, for example, assessing the impact of CSF loss [43]. However, in that case the computational model was simplified to include a coarse mesh of the brain itself, with no surrounding structures. While similar in application, the present study differs in objective to Clatz [43] and subsequent works [44] in the desire to model the small-scale effects of head re-orientation and derive material properties, with no intraoperative data.

Within existing computational models geometric detail varies, with groups including different combinations of selected brain regions, the dural septa [37], grey/white matter distinction [38], bridging veins [39] and numerous representations of CSF. These range from low shear modulus, incompressible solid elements to rigid connections or sliding interfaces [34]. Only recently have these included element/fluid–structure interaction (FSI) formulations, treating the CSF as a Newtonian fluid [28]. Efforts have been made to study the impact of the PAC in impact scenarios [22,45]; however, to the best of the authors' knowledge, this study is the first to include a combined spring/FSI representation of this space.

This approach was found to be critical in recreating realistic displacements across the entire brain, as in earlier development using solid elements it was found to be challenging to recreate accurately the tethering effect of the arachnoid trabeculae while allowing compression on the opposing side of the brain. Grey/white matter distinction and material anisotropy was not included in this model. However, this is unlikely to have a significant impact under this loading regime, as the level of anisotropy is known to be limited [15], deviatoric stresses are very low and the grey and white matter have similar properties [15].

### 4.2. Material properties and parametric analysis

The present study used GPR to facilitate the inverse modelling of material properties from sparse training data. ‘Residual error’, or the magnitude of discrepancy between the human displacement fields and those generated by the computational model, was used to quantify the suitability of each set of parameters tested. This yielded a scalar descriptor, which, when minimized over 24 300 000 test sets, yielded the apparent optimum combination of parameters. This approach was highly effective in allowing systematic evaluation of a very large number of parameter sets, and also enabled the sensitivity and uniqueness of the optimum parameters to be evaluated. To do this by running models for every parameter set would have been prohibitively time consuming. Future work assessing the applicability of other inverse methods [46] to the same training data would be a meaningful addition.

Numerous constitutive models have been developed to capture the unique properties of the brain, each better or worse under specific loading scenarios. These can use between 3 and 20 material parameters [47]. It was hypothesized that a single-phase, compressible formulation would be suitable, as the dynamic response was not desired. The Ogden constitutive model is often suggested when considering the elastic response of brain tissue [47]. Furthermore, the relatively simple formulation with only three material parameters and successful implementation in some of the prominent studies within the field [15,24,48] provided sufficient grounds to use the Ogden model for this study.

The deviatoric stiffness response of the modified one-term Ogden formulation [15] is driven by two parameters: the initial shear modulus (*μ*) and exponential coefficient (*α*) which controls the increase in stiffness at large strains. Numerous previous works have attempted to identify these parameters through mechanical testing; some notable examples include:

— Miller & Chinzei [24]—*μ* = 842 Pa, *α* = −4.7
— Prange & Margulies [48]—*μ* = 296 Pa, *α* = 0.0323
— Budday *et al*. [15]—*μ* = 350 to 1430 Pa, *α* = −25.3 to − 19.0
— Mihai *et al*. [49]—*μ* = 378 Pa, *α* = −8.05
— Present study—*μ* = 670 Pa, *α* = −19

Considering that results from the present study were obtained through an *in vivo*, inverse modelling approach, the similarity of the results to previous *ex vivo* mechanical testing is encouraging. The recent regional analysis by Budday *et al*. [15] considered samples from the corpus callosum, corona radiata, basal ganglia and the cortex. The model was fitted to combined loading, yielding mean shear modulus values of 350, 660, 700 and 1430 Pa, respectively. Here, regional tissue distinction was not included in the model; however, the obtained shear modulus of 670 Pa appears to be an appropriate value when this regional distinction is not made. Other attempts to obtain material properties *in vivo* using MRE have found the shear modulus to be of the order of 3 kPa [50]. This apparently higher stiffness is most likely due to high strain rates in MRE, further highlighting difficulties in applying values identified through this method to low-strain-rate applications. There is perhaps less agreement in the literature regarding the exponential coefficient. This perhaps stems from a lack of sensitivity to this parameter under low strains. However, once again, the value derived here falls comfortably within the previously published range.

It is now well established that the incompressibility assumption is only valid in certain circumstances [12,47]. During mechanical testing, Budday *et al*. [15] found a clear pre-conditioning effect over three consecutive cycles which recovered after soaking the sample with phosphate-buffered saline solution. This suggests that the effect is down to fluid loss, rather than damage to the tissue itself. When not concerned with time-dependency, the bulk modulus or Poisson's ratio can be used to account for volume change within a material [51]. Here, the bulk modulus was determined to be 148 kPa. At low strains, the Poisson's ratio *ν* can be found according to equation (4.1):

4.1 $$\nu =\frac{3k-2\mu }{2(3k+\mu )},$$

yielding a result of 0.4977. Assessment of the bulk modulus directly in the literature has been limited. Values for the bulk modulus of the order of 40 000 Pa (*ν* ∼ 0.4) were initially reported [52–54], but are thought to be incorrect due to methodological assumptions. In direct consolidation testing of real human tissue, Franceschini *et al*. [12] determined a drained Poisson's ratio of 0.496, with full consolidation occurring with volumetric strain of the order of 3%.

The lack of attention given to the bulk modulus in most modelling implementations may give the impression that choosing an accurate value is unimportant. The value is often varied inexplicably [55], with an in-depth review of the literature by Morin *et al*. [56] reporting values for the Poisson's ratio to be 0.4 ≤ *ν* ≤ 0.495. When of the order of 500 000 Pa (*ν* ∼ 0.499) and up, brain shift is limited to small scale, rigid body displacement. On the other hand, much lower values lead to unrealistically large displacements. The obtained value of 0.4977 supports the findings of Franceschini *et al*. [12], especially when considering that the volumetric strains found here peaked at 0.7%, suggesting full consolidation was not achieved. Although the difference between 0.499, 0.496 and 0.49 appears small in terms of Poisson's ratio, it is significant in terms of bulk modulus. This study confirms that in confined loading scenarios such as positional brain shift, this is very influential and needs more in-depth investigation.

Until recently, the PAC has been significantly under-researched. Direct measurement by Jin *et al*. [57–60] estimated the minimum tensile stiffness to be around 61 kPa, increasing with increasing strain rate. Mazumder *et al*. [61] used inverse modelling methods based on indentation of sheep brain, yielding a value of approximately 24 MPa (when converted). However, others have suggested that the methodology employed has significant limitations [34]. More recently, Benko *et al*. [62] conducted *in situ* mechanical testing on human brains using optical coherence tomography, thus avoiding the difficulties associated with mechanical testing of this thin structure. They report [62] a mean traction modulus of 12.6 ± 4.8 kPa, which compares well with the value of 7.7 kPa found here. Interestingly, Benko *et al*. also report that PAC stiffness is greater by approximately 21% in superior regions, presenting an opportunity for future development of the model. Finally, reported values from the pia mater range from 0.5 to 20 MPa [25,26,34]. Although sensitivity to this parameter was low, the derived value of 1.2 MPa falls within this range and is likely to be at the lower end based on recent research [26]. Here the PAC was defined to have a constant thickness of 15 µm; any variation would impact the apparent stiffness of the structure, potentially explaining some of this deviation.

Additional sensitivity analysis was undertaken using the GEM-SA software, identifying the bulk modulus as the most influential parameter for volume-constrained, gravity-induced deformation of the brain. Noting the log scale used in figure 2, the error ratio can be seen to increase rapidly on either side of the optimum value, supporting the results of the sensitivity analysis. The shear modulus of the brain was found to be more influential than the exponential stiffening coefficient, probably due to the relatively low strains. Joint effects were significant, highlighting the fact that the biomechanical process is complex. By contrast to some previous works concerning neurosurgery [63], the results presented here suggest that accurate representation of the material properties of the brain is essential in achieving realistic deformation, at least at small strains under quasi-static loading.

### 4.3. Biomechanics

This study adds to the relatively small body of work focusing on non-rigid brain–skull deformation due to positional effects alone. Early work by Hill *et al.* [5] found positional brain shift to be less than 1 mm. More recently, Schnaudigel *et al*. [6] found the displacement to be maximal in central structures, with a magnitude of 0.6–1.3 mm. By contrast, Monea *et al*. [7] found cortical deformations up to 7.86 mm, although this seems unlikely given the width of the subarachnoid space. This study found deep brain displacement of the order of 1 mm, supporting the findings of Hill *et al*. and Schnaudigel *et al*.

Extension of the springs on ‘top’ surface in either orientation was limited, with ‘bottom’ springs under little to no load. This suggests a tethering effect, limiting the surface displacement of the brain which would otherwise occur due to the marginally different densities of the brain and CSF (the resultant force on the brain being of the order of 5 N). Low sensitivity to the stiffness of the pia mater suggests that this structure has a small mechanical contribution, although overestimating the stiffness still leads to high levels of residual error, suggesting that the use of an arbitrary value would be ill-advised.

**Figure 5.**
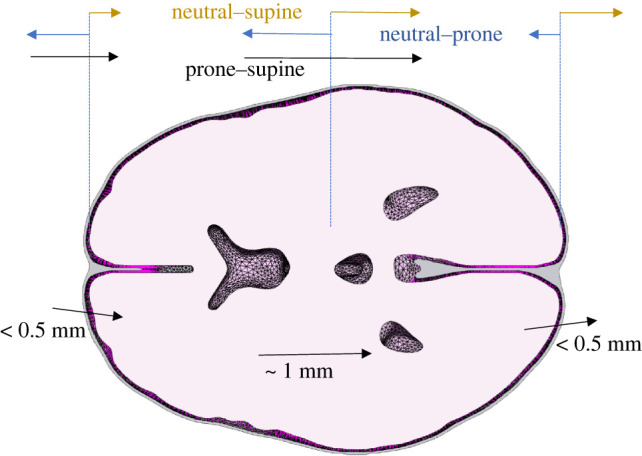
Schematic of the extension and compression of the PAC in neutral–prone/supine and the resulting supine–prone displacement. Even with relatively low stiffness, the limits displacement on the ‘top’ surface with respect to gravity in each orientation, meaning boundary displacement is largely driven by ‘sagging’ on the opposing side.

It is proposed that positional brain shift has two components: a small component of rigid body translation (approx. 0.5 mm), governed largely by the stiffness of the PAC, and deep structure ‘sagging’ (approx. 1 mm), governed predominantly by the volumetric stiffness of the brain. A schematic of this can be found in figure 5. While the amount of deformation may be inconsequential for some clinical scenarios, clinicians undertaking high-precision procedures may wish to consider the potential impact of positional brain shift.

While the results are encouraging, this study has limitations. The pre-stress in the intrancranial tissues is unknown; this is particularly relevant to the arachnoid trabeculae. In an attempt to overcome this, the model geometry was modified to maintain a uniform PAC thickness and exposed to ‘neutral’–prone and ‘neutral’–supine loading. While this is not what happens in reality, it was considered to be a lesser assumption than accurate PAC segmentation and estimation of pre-stress. The brain tissue itself is heterogeneous [15] and this was not replicated in the model. Finally, the derivation of the subject displacement fields was imperfect. This was due to spatial warping of the underlying MR images, inaccuracies at boundaries and displacements of the order of the voxel size. Erronous displacements around the surface may have affected the optimized model.

## 5. Conclusion

To the authors’ knowledge, this is the first attempt to derive *in vivo* material properties of the brain and PAC using gravitational loading and a computational model. A biofidelic FE model has been developed, using a combined fluid and discrete spring representation of the PAC, allowing for proper representation of tensile and compressive properties. From this, it was identified that relatively limited volume change, controlled by the bulk modulus of the brain, plays an important role in quasi-static deformation. Material parameters, in particular the bulk modulus of the brain, should be chosen carefully, especially when the application is volume-constrained. Values of 148 kPa, 670 Pa, −19, 1210 kPa and 7.7 kPa for the brain bulk modulus, shear modulus and exponential coefficient, pia mater Young's modulus and out-of-plane Young's modulus of the PAC, respectively, were found. In future, this approach could be used to assess variation across different patient groups, or the impact of geometric variation between subjects. More significantly, the accurate pre-operative prediction of gravity-induced brain shift could allow for adjustment to surgical plans, improving the safety and efficacy of stereotactic neurosurgical procedures.

## Data Availability

Data can be accessed via the following link: https://osf.io/fc3wm/?view_only=70e7dd7765034eab8c850e93e1499966.

## Appendix A

See table 3.

**Table 3.** The obtained optimum values obtained for each of the 11 subject datasets for each tested parameter. As it is not mathematically rigorous to take the mean of the individual subject results, the values calculated from the averaged displacement field are included for reference.

subject ID	baseline ‘error’ (mm)	brain			pia mater	PAC
		bulk modulus (kPa)	shear modulus (Pa)	exponential coefficient	Young's modulus (kPa)	spring stiffness (N m^−1^)
1	0.60	91	525	−16	460	6.30
2	0.66	143	695	−19	1440	8.0
3	0.52	143	695	−19	1300	8.0
4	0.49	107	1075	−30	3660	12.3
5	0.56	91	525	−17	510	5.5
6	0.67	91	525	−17	510	5.5
7	0.58	153	695	−20	1440	14.0
8	0.62	138	695	−19	1300	7.2
9	0.43	138	695	−19	1300	8.0
10	0.41	143	695	−19	1440	8.0
11	0.53	138	695	−19	1300	8.0
average	*0**.**46*	*148*	*670*	*−19*	*1210*	*8**.**0*
